# Isolation of Circulating Tumor Cells from Peripheral Blood Samples of Cancer Patients Using Microfluidic Technology

**DOI:** 10.17691/stm2020.12.6.08

**Published:** 2020-12-28

**Authors:** A.B. Volovetskiy, P.A. Malinina, A.Y. Kapitannikova, S.V. Smetanina, I.A. Kruglova, A.V. Maslennikova

**Affiliations:** Researcher, Laboratory of Nanotheranostics, Institute for Molecular Medicine, Biomedical Science and Technology Park; I.M. Sechenov First Moscow State Medical University (Sechenov University), 8/2 Malaya Trubetskaya St., Moscow, 119991, Russia;; Clinical Resident, Department of Oncology, Radiation Therapy and Radiation Diagnostics; Privolzhsky Research Medical University, 10/1 Minin and Pozharsky Square, Nizhny Novgorod, 603005, Russia;; Researcher, Laboratory of Nanoteranology, Institute for Molecular Medicine, Biomedical Science and Technology Park; I.M. Sechenov First Moscow State Medical University (Sechenov University), 8/2 Malaya Trubetskaya St., Moscow, 119991, Russia;; Head of the Сytological Laboratory; Nizhny Novgorod Regional Oncologic Dispensary, 11/1 Delovaya St., Nizhny Novgorod, 603163, Russia;; Physician of Clinical Laboratory Diagnostics; City Hospital No.35, 47 Respublikanskaya St., Nizhny Novgorod, 603089, Russia;; Head of the Department of Oncology, Radiation Therapy and Radiation Diagnostics; Privolzhsky Research Medical University, 10/1 Minin and Pozharsky Square, Nizhny Novgorod, 603005, Russia; Professor, Department of Biophysics National Research Lobachevsky State University of Nizhni Novgorod, 23 Prospekt Gagarina, Nizhny Novgorod, 603950, Russia

**Keywords:** circulating tumor cells, disseminated tumor process, microfluidic technologies, fluid biopsy, immunocytochemical study, fluorescent antibodies, tumors of epithelial origin, circulating tumor clusters

## Abstract

**The aim of the investigation** was to study the potential of an innovative microfluidic technology for the isolation of circulating tumor cells (CTCs) from the peripheral blood samples of cancer patients.

**Materials and Methods.:**

Peripheral blood samples from 5 patients with disseminated malignant tumors of epithelial origin were processed with the use of the microfluidic technology (based on a specifically designed silicone chip). The cells were separated according to their density criterion based on the lateral migration of solid particles in a liquid due to inertial forces. With the help of the designed chip configuration, the cells over 13 μm in size which is larger than the standard size of blood cells were isolated. The resulting target cell fraction was stained by the Romanowsky–Giemsa method. Staining with the fluorescent Anti-Cytokeratin (CK3-6H5)-FITC antibody was carried out to confirm the epithelial nature of the cells, and the DAPI dye was used to contrast the nucleus. The blood of a healthy volunteer and tumor cells of the A549 line were used for the immunocytochemical studies.

**Results.:**

The tumor cells in peripheral blood (in the number of 1 to 9) were detected in all 5 patients. CTC clusters of 2–5 cells were identified in blood samples from the patients with laryngeal cancer, non-small cell lung cancer, and floor of the mouth cancer. A bright saturated staining of the A549 tumor cells was obtained using the Anti-Cytokeratin (CK3-6H5)-FITC antibody, corresponding to the staining of the cytoskeleton of epithelial cells. Successful nuclear staining with DAPI confirmed that the isolated target cells are not damaged during microfluidic separation.

**Conclusion.:**

The microfluidic technology that has been used enables effective intact CTCs isolating from the peripheral blood of cancer patients. The epithelial nature of the isolated cells can be confirmed by immunocytochemical studies.

## Introduction

The ability to metastasize is one of the key features of malignant tumors. The program of the metastatic cascade includes successively loss of cell adhesion of the primary focus (site), their migration from the primary focus, activation of the epithelial-mesenchymal transition, intravasation, circulation in the blood flow with subsequent spread through the bloodstream and is completed with extravasation and proliferation with the formation of a secondary metastatic focus (site) [1]. These “steps” of the metastatic cascade are connected by a unique state of a tumor cell — circulation and survival in the systemic circulation. Some of these cells “settle” in the organs forming a pool of dormant cancer cells, another part continues to circulate in the peripheral blood and forms a pool of circulating tumor cells (CTCs) [2]. CTCs were first discovered and documented by the Australian pathologist T.R. Ashworth more than a century ago [3], however, the understanding of the clinical, as well as the prognostic and predictive significance of CTCs, has only emerged in the last decade. The histological examination of biopsy specimens of a primary and/or metastatic tumor makes it possible to identify its molecular profile and determine adequate drug therapy.

This information may be insufficient in light of the existing evidence of temporal and spatial heterogeneity of a neoplasm, particularly that arising from the selection of tumor clones “under the pressure” of antitumor therapy [4]. A repeated biopsy procedure of a tumor focus is not always possible or can be technically difficult if it is localized in internal organs. Thus, CTCs are the most convenient objects for dynamic study, since they can be obtained by simple collection of the patient’s peripheral blood. They represent a dynamically changing fraction of cells, thereby being the key to understanding tumor heterogeneity, biology of metastasis, and opening up the possibility of controlling tumor evolution. The problem of isolating CTCs from a blood sample and their subsequent characterization has not yet been fully resolved, which is one of the main obstacles to the realization of their prognostic potential in the clinic.

Basically, all methods for isolating CTCs can be divided into biological and physical ones. The biological methods are based on the ability of tumor cells to interact with specific antibodies. Currently, this approach is considered as the gold standard for the isolation of CTCs with an epithelial phenotype, characterized by high reproducibility and specificity, and the corresponding CellSearch technology is approved for clinical use by the Food and Drug Administration (FDA) [5]. Its main shortcoming is the impossibility of separating cells that have lost their epithelial phenotype due to the epithelial-mesenchymal transition, and which are believed to play a key role as a driver of the metastatic process [6].

The operation of the system is based on the indicated above ability of tumor cells. In the system, a functionalized tip enriched with monoclonal antibodies to epithelial cell adhesion molecules — EpCAM is inserted into a standard Seldinger catheter, which allows the system to connect directly to the bloodstream of a patient with a large blood volume analysis, due to which EpCAM-positive CTCs are captured and retained at the tip [7, 8].

An alternative to positive selection is negative isolation techniques based on the binding of leukocytes to specific antibodies (CD45) and their subsequent removal from the analyzed sample (Label-free [9] and RosetteSep [10]). A significant advantage of this selection is the ability to isolate CTCs regardless of their immunophenotype, as well as the preservation of these cells viable and suitable for further research and/or cultivation [11].

Biological methods of CTC selection also include their counting and isolating using fluorescence-activated cell sorting (FACS) via flow cytometry. This method makes it possible to obtain cell clones of high purity but requires antibody selection and a large starting amount of material [12, 13]. The disadvantages of the method also include the complexity of the simultaneous assessment of a large number of parameters (8–12) due to the overlap of the fluorescence spectra and a rather high cost [14].

Magnetic-activated cell sorting (MACS; Miltenyi Biotec, Germany) differs by the use of conjugates of specific antibodies to the target cell fraction with supermagnetic nanoparticles, which allows separation in a directed magnetic field. The technology is quite simple, but it is characterized by a high cost of the system and consumables. Moreover, the efficacy of the method depends on the affinity of the selected antibodies to the target protein as well as it does for any other biological separation method [15].

The methods for physical selection of CTCs are based on the difference in the physical properties of tumor cells from those of normal ones: their size, density, and mechanical properties (rigidity, deformation capacity). Selection based on differences in the size of normal and tumor cells allows them to be separated by simple microfiltration since blood cells are usually smaller in size compared to tumor cells [16, 17]. The method is simple to implement and apply and relatively cheap, but it also has significant shortcomings: there is a probability of skipping small-sized cancer cells, the filter is easily clogged and can only be used for the analysis of a limited blood volume.

Cell separation during centrifugation in accordance with cell density [18, 19] carries the risk of losing individual cells as a result of aggregate formation and has a low sensitivity since cancer cells are heterogeneous and not identical in their physical properties.

The most promising method for isolating CTCs from peripheral blood seems to be the use of microfluidic systems, which are based on lateral migration of solid particles in a liquid due to inertial forces [20, 21]. This enables the isolation of tumor cells larger than normal blood cells. The disadvantages of this method include a large number of “background” leukocytes, which complicates the enumeration and characterization of isolated CTCs, as well as the high cost of consumables [22–28]. At the same time, the microfluidic chip itself has a relatively low cost, requires a minimum volume of blood samples, and allows the separation of tumor cells regardless of their phenotype, which provides a significant advantage of this system over the CellSearch system certified for clinical use.

Until now, the choice of a method for the separation of CTCs from peripheral blood for applying in routine clinical practice with minimal losses and maximum convenience remains controversial. In our study, in order to isolate CTCs from the peripheral blood flow, we used a microfluidic technology based on a silicon chip of a certain configuration.

**The aim of the investigation** was to study the potential of the innovative microfluidic technology for the isolation of circulating tumor cells from peripheral blood samples of cancer patients and their subsequent characterization.

## Materials and Methods

The study was carried out on the basis of the chemotherapy department of the polyclinic of the Nizhny Novgorod Regional Clinical Oncologic Dispensary (day hospital). We studied the samples of peripheral blood from 5 patients with disseminated malignant neoplasms of epithelial origin, who received systemic treatment due to the progression of the disease with the development of a metastatic process. The study was carried out in accordance with the Declaration of Helsinki (2013) and approved by the Ethics Committee of the Nizhny Novgorod Regional Clinical Oncologic Dispensary. Voluntary informed consent was obtained from each patient. General information about the subjects is presented in the “Results” section.

Inclusion criteria: established diagnosis of disseminated malignant neoplasm of epithelial nature (cancer); histological verification of the primary tumor and secondary metastatic foci; functional status on the ECOG scale 0–2. The exclusion criterion was functional status on the ECOG scale 3–4.

### Preparation of blood samples.

Peripheral blood samples from the selected patients were taken from the cubital vein into disposable sterile vacuum tubes treated with EDTA-K3. To exclude the ingress of epithelial cells into the sample after skin puncture, the first milliliter of the patient’s blood was disposed of, and then 9 ml of blood was taken. Within no more than 3 h after collection, the blood was treated with lysis buffer at room temperature (MACS Separation Buffer; Miltenyi Biotec) in the blood to buffer ratio of 1.0:2.5 for 20 min by constant stirring in order to destroy the erythrocyte fraction. Then the blood lysate was centrifuged (CM-6M; ELMI, Latvia; 5 min, 500 g), the supernatant was disposed of, and the sediment containing the target CTCs was diluted with phosphate buffered saline (PBS) up to 10 ml.

### Description of the microfluidic chip and cell separation technology.

**Figure 1:**
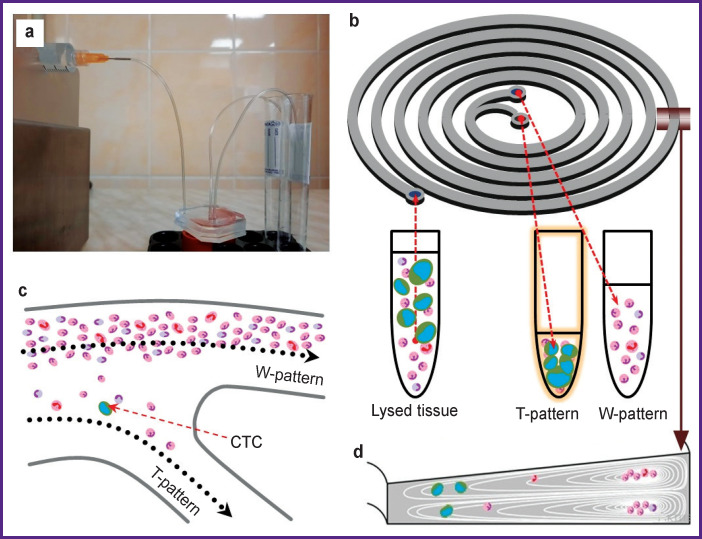
Installation for separation: (a) appearance; (b) a schematic representation of a silicone microfluidic chip; (c) a fork in the center of the chip; (d) cross-section of the turn cavity of the chip with the inside distribution of the particles depending on their size

The next stage of the study was the separation of the resulting suspension using a syringe pump (SpLab01; Vilitek, Russia) and a silicon spiral microfluidic chip (Figure 1).

The syringe with the obtained cell suspension was installed in the syringe pump and a constant outflow rate (1.7 ml/min) was set up. The cell suspension entered the chip from the syringe through a plastic tube with the inner diameter of 0.5 mm (Figure 1 (a), (b)). Inside the chip, the cells were separated according to their density based on the lateral migration of solid particles in a liquid due to inertial forces (Figure 1 (c), (d)). As a result, the overwhelming number of cells less than 13 μm in size, in accordance with the principle of operation of the chip, were separated into the “trash” test tube (hereinafter referred to as a W-sample), and the overwhelming number of the cells larger than 13 μm were separated into the “target” test tube (hereinafter referred to as a T-sample). It should be noted that the T-sample contains a significant number of leukocytes, which requires additional verification of the cells in the T-sample.

### Detection of circulating tumor cells in the targeted samples.

 The staining of the blood samples from 5 patients was performed by the Romanowsky– Giemsa method. The sedimentation of the cells from the CTC-enriched suspension was performed using an Awel C12 cytocentrifuge (Domel, Slovenia) for 9 min at 500 g onto a glass slide, after that the samples were fixed by the May–Grünwald method for 3 min. The fixed blood smears were placed in the Romanowsky–Giemsa working solution, after 30 min, the preparations were washed with running water for 1–2 s and air-dried.

### Immunocytochemical staining with Anti-Cytokeratin-FITC and DAPI dyes.

To try out the method of CTC characterization, 5000 cells of the A549 (catalog number ATCC® CCL-185™) human lung carcinoma cell line were added to 9 ml of blood samples of a healthy volunteer and separated by the above method. The cells were cultured in the DMEM (PanEсo, Russia) medium containing 2 mmol L-glutamine (PanEco) and 10% fetal calf serum (HyClone, USA), at 37°C and 5% CO_2_, in culture flasks with an area of 25 cm^2^. At each stage of cell passaging, the cells were treated with 0.25% trypsinversene solution (PanEco). Subculturing was performed 2–3 days after the culture reached 80% confluence [29].

The sedimentation of the cells from the CTC-enriched suspension was performed using an Awel C12 cytocentrifuge (Domel) for 9 min at 500 g onto a glass slide after which the obtained preparations were fixed at room temperature in the Triton X-100 paraformaldehyde medium (1%) for 7–10 min. Then the preparations were washed in PBS and dried. To block nonspecific binding, the preparations were incubated with 5% bovine serum albumin (BSA) in PBST (0.1% Tween in PBS, pH 7.2) for 90 min at room temperature in the humid medium and slightly dried. After that, the solution of the labeled Anti-Cytokeratin (CK3-6H5)-FITC (Miltenyi Biotec) antibody diluted with a 1:50 ratio was put on the glass slide with the sample and incubated for 4 h at room temperature in the humid environment. After that, the obtained samples were treated with the DAPI (4’, 6-diamidino-2-phenylindole dihydrochloride) dye, followed by exposure for 10 min and washing in PBS. The use of DAPI (nuclear staining) in this study was due to the need for additional verification of the integrity of the identified cells.

The choice of the Anti-Cytokeratin (CK3-6H5)-FITC fluorescent antibody was primarily due to the need to identify signs of the epithelial affiliation of the isolated cells, which will definitely indicate their tumor nature. In addition, the use of the FITC label makes it possible to distinguish the received fluorescent signal from DAPI. At the final stage, the preparations were dried and fixed with a mounting medium (ProLong AntiFade Reagent; Thermo Fisher Scientific, USA). The obtained preparations were stored in foil in the dark at a temperature of 4°C.

### Microscopic examination.

**Figure 2:**
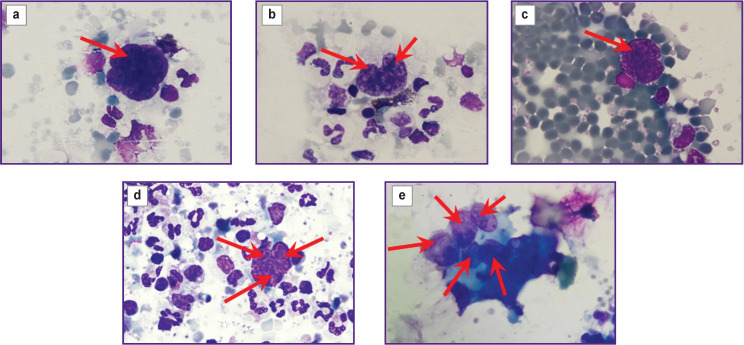
Images of atypical cells in the blood samples of the studied patients: (a) with cancer of the mandibular alveolar process; (b) with laryngeal cancer; (c) with thyroid cancer; (d) with non-small cell lung cancer; (e) with floor of the mouth cancer. Romanowsky–Giemsa staining; ×100. The arrows indicate atypical cells in the “targeted” blood samples after separation in the microfluidic chip

**Figure 3:**
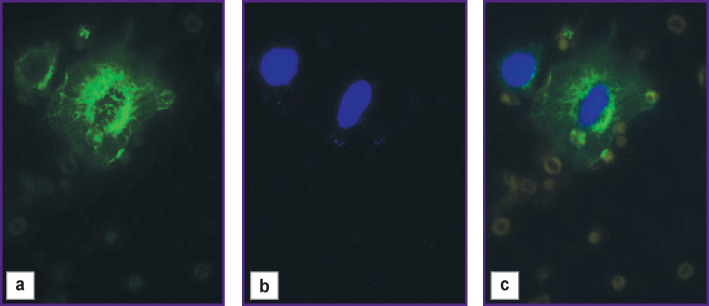
Cells with atypical morphology obtained from a peripheral blood sample of a healthy volunteer with the preliminary addition of the A549 cell line: (a) staining with Anti-Cytokeratin (CK3-6H5)-FITC fluorescent antibodies; fluorescence excitation — 488 nm; signal reception — 525 nm; (b) nuclei contrasting with DAPI; excitation of fluorescence — 405 nm; signal reception — 470 nm; (c) overlay of Anti-Cytokeratin (CK3-6H5)-FITC and DAPI

Photographic (Figure 2) and fluorescent (Figure 3) images were obtained using a LSM 710 DUO confocal laser scanning microscope on the Axio Observer Z1 inverted tripod (Carl Zeiss, Germany). The fluorescence excitation of Anti-Cytokeratin (CK3-6H5)-FITC was performed at a wavelength of 488 nm, the signal was recorded at 525 nm. DAPI fluorescence was excited at a wavelength of 405 nm, and the signal was recorded at 470 nm.

## Results and Discussion

Tumor cells in the peripheral blood were detected in all 5 patients. The number of identified CTCs in the patients involved in the study is presented in the table. CTC clusters of 2–5 cells were identified in the blood samples from the patients with laryngeal cancer (Figure 2 (b)), non-small cell lung cancer (Figure 2 (d)), and floor of the mouth cancer (Figure 2 (e)).

**Table:** Patient information and the number of circulating tumor cells isolated from the peripheral blood samples

Patient (age, sex)	Diagnosis, ECOG status	Number of CTCs in the sample
No.1, aged 69, male	Carcinoma of the oral mucosa (mandibular alveolar process on the right) — cT_4a_N_2c_M_1_ (PULM), stage IV C. Clinical group II. Histology report: moderately differentiated squamous cell carcinoma, ECOG 1	5
No.2, aged 54, male	Laryngeal cancer — cТ_3_N_2b_M_1_ (OSS), stage IV C. Clinical group II. Histology report: poorly differentiated squamous cell carcinoma, ECOG 1	2 cells in the cluster
No.3, aged 37, female	Thyroid cancer cТ_3_N_1b_M_1_ (HEP), stage II. Clinical group II. Histology report: papillary cancer, ECOG 1	1
No.4, aged 52, male	Non-small cell lung cancer (mediastinal form) — cT_3_N_x_M_1_ (OSS), stage IV. Clinical group II. Histology report: poorly differentiated squamous cell carcinoma without keratinization, ECOG 2	9, including 2 clusters of 2 and 3 cells, respectively
No.5, aged 67, female	Floor of the mouth cancer — cT_2_N_2_M_1_ (PULM), stage IV C. Clinical group II. Histology report: moderately differentiated squamous cell carcinoma, ECOG 1	7, including a cluster of 5 cells

At the next stage, immunocytochemical phenotyping of epithelial cells was performed. The study was performed on the A549 lung carcinoma cell line, previously added to a peripheral blood sample of a healthy volunteer and separated by the above method. A bright saturated staining of cells with atypical morphology was obtained using Anti-Cytokeratin (CK3-6H5)-FITC antibodies, corresponding to the staining of the cytoskeleton of epithelial cells (Figure 3 (a)). Successful nuclei staining with DAPI (Figure 3 (b)) showed the integrity of the identified atypical cells (the cells isolated in the T-sample are not damaged during separation).

Thus, in this study, the method of CTC separation from peripheral blood samples using a microfluidic chip was tried out in the clinic. With this technology, CTCs have been found to be isolated in patients with disseminated tumor process in the number comparable to the previously obtained data [26]. The main problem when working with a microfluidic chip was a large number of “junk” white blood cells in the final sample, which complicates the identification of tumor cells and requires further improvement of the technology in order to achieve a higher quality separation of the “target” and “junk” samples. The further research involves working with larger blood samples (20 ml) and searching for ways to reduce the cost of the technology.

The immunocytochemical study with antibodies to cytokeratin confirmed the epithelial nature of the isolated cells. When Anti-Cytokeratin (CK3-6H5)-FITC antibodies (antibodies conjugated to a fluorescent label) interact with tumor cells, specific binding of cytoskeletal elements occurs, which makes it possible to detect and distinguish these cells from blood cells by laser scanning microscopy.

In the experiments, the burnout of the FITC fluorescent label within a day was noted, which requires its replacement in further studies.

Confirmation of the epithelial nature of cells found in peripheral blood is important, but not always informative enough to characterize all identified CTCs due to the phenomenon of epithelial-mesenchymal transition. CTCs, as well as tumor cells in general, are characterized by population heterogeneity, and a logical next step would be to select additional antibodies for a more complete characterization of the isolated cell pool.

## Conclusion

The technology of immunocytochemical staining of circulating tumor cells makes it possible to effectively isolate these cells from the peripheral blood of cancer patients; however, an improvement of the verification technique of the obtained cells is needed including the use of more stable fluorescent antibody-bound labels (Alexa fluor 488, 568, 647).
